# Intraocular pressure change with face-down positioning after macular hole surgery

**DOI:** 10.1371/journal.pone.0242567

**Published:** 2020-11-17

**Authors:** Sung Won Choi, Chong Eun Lee, Yu Cheol Kim

**Affiliations:** Department of Ophthalmology, Keimyung University School of Medicine, Daegu, Republic of Korea; University of Toronto, CANADA

## Abstract

This study evaluated changes in intraocular pressure (IOP) with face-down positioning (FDP) following surgical treatment of idiopathic macular hole. We retrospectively reviewed the records of 130 patients diagnosed with idiopathic macular hole who underwent pars plana vitrectomy (PPV) with intravitreal gas injection after fluid-gas exchange. We analyzed IOP changes in both eyes following FDP over the course of 7 days. The mean IOP of the operated eyes was 14.98±2.95 mmHg preoperatively and 16.82±3.12 and 15.57±6.10 mmHg on postoperative days 2 and 7, respectively. In contralateral eyes, the mean IOP changed from 14.78±3.15 mmHg preoperatively to 16.27±1.87 and 14.40±4.14 mmHg on postoperative days 2 and 7, respectively. On postoperative day 2, the IOP increased in both eyes compared to the preoperative values, but the increase was significant only in operated eyes (*p* = 0.039). In contralateral eyes, the IOP on postoperative day 7 was significantly decreased compared with that on postoperative day 2 (*p* = 0.021) and in eyes with an axial length ≥ 23.0 mm, compared with the preoperative values (*p* = 0.042). The IOP of the operated eyes on postoperative day 7 was higher than that of the contralateral eyes (*p* = 0.039). Based on a short-term follow-up, FDP after PPV with intravitreal gas tamponade for the treatment of idiopathic macular hole may cause IOP elevation not only in the operated, but also in the contralateral eyes; the IOP increase in contralateral eyes was not as significant as that in operated eyes and was not maintained over 7 days after surgery; the IOP change seems to be affected by axial length and lens status.

## Introduction

Idiopathic macular hole is a full-thickness defect of the retinal neurosensory layer caused by vitreous traction. Pars plana vitrectomy (PPV) combined with intravitreal gas tamponade and internal limiting membrane peeling is considered the standard treatment [1]. Since Kelly and Wendel [2] reported that face-down positioning (FDP) was necessary following macular hole surgery, it has become a routine postoperative procedure. Even though there is increasing evidence that short-term or no FDP may be equally effective, occasional cases were believed to need long-term FDP to close macular holes [3–5]. Regardless, controversy exists concerning its necessity and duration as FDP may induce complications, such as intraocular pressure (IOP) increase, thrombosis, and embolism [6–9]. In particular, FDP can cause anterior movement of the iris-lens diaphragm, leading to narrowing of the anterior chamber angle, which causes a pupillary block and consequently increasing IOP. It has thus been used as a provocation test for angle-closure glaucoma [10].

Elevated IOP has been previously reported with FDP in operated eyes; however, few studies have investigated IOP changes in the contralateral eye after macular hole surgery. Lee and Kim [11] reported that bilateral acute angle-closure glaucoma developed after macular hole surgery and FDP was the single most important cause of IOP increase in both eyes. Therefore, we evaluated the IOP changes and related influencing factors both in the operated and contralateral eyes after PPV followed by FDP for idiopathic macular hole.

## Materials and methods

We retrospectively reviewed the medical records of patients who underwent PPV with gas tamponade for the treatment of idiopathic macular hole from January 2010 to May 2018. This study was approved by Keimyung university Dongsan hospital Institutional Review Board (2018-11-038) and adhered to the tenets of the Declaration of Helsinki. All data were fully anonymized before we accessed them and the Institutional Review Board waived the requirement for informed consent.

Patients who had undergone intraocular surgery (other than uneventful cataract surgery in either eye) before macular hole surgery were excluded. Furthermore, we excluded patients with a history of refractive surgery, intraocular laser procedures, diabetic retinopathy, ocular trauma, a preoperative IOP exceeding 22 mmHg, or diagnosis of glaucoma in either eye.

Both eyes were examined before surgery, including best corrected visual acuity assessment, IOP measurement using a Goldmann applanation tonometer, slit lamp microscopy, and fundus examination. The macular hole was confirmed by optical coherence tomography (OCT) using swept-source OCT (DRI-OCT Triton, Topcon, Japan) or spectral domain OCT/scanning laser ophthalmoscopy (OTI, Ophthalmic Technology, Toronto, Ontario, Canada). Axial length was measured using an Axis II A-scan (Quantel Medical Inc., Bozeman MT, USA).

Macular hole surgery was performed by a single surgeon (YCK) using a standard three-port PPV, internal limiting membrane removal and complete fluid-gas exchange with shaving of the peripheral vitreous, followed by intravitreal gas injection using 20% sulfur hexafluoride gas or 12% perfluoropropane. From postoperative day 1, topical antibiotics (0.5% Moxifloxacin Hydrochloride, Vigamox^®^, Alcon, Fort Worth, TX, USA) and topical steroids (1% Prednisolone Acetate, Pred forte^®^, Allergan Inc., Irvine, CA, USA) were applied to the operated eye every two hours. All patients were instructed to maintain FDP for 7 days after surgery, and bilateral IOP was measured by Goldman applanation tonometry (GAT) on postoperative days 2 and 7. All patients maintained a sitting position for 5 minutes before measurement. If the IOP of the operated eye exceeded 22 mmHg, topical timolol/brimonidine fixed combination agent (Combigan®, Allergan Inc., Irvine, CA, USA) was applied.

The IOP changes of the non-operated eye as well as the operated eye at each postoperative follow-up were evaluated because otherwise it would be difficult to rule out that the increase in IOP was related to surgery itself. Continuous variables are expressed as the mean ± standard deviation. Variables were compared between groups using either an independent samples *t*-test or Mann-Whitney U test. Within-group comparisons were performed using a paired *t-*test, Kruskal-Wallis test, or Wilcoxon’s sign rank test. A *p*-value less than 0.05 was considered statistically significant. Statistical analyses were performed using IBM SPSS Statistics version 22 (IBM Corp., Armonk, NY, USA).

## Results

A total of 130 patients (mean age, 67.43±8.15 years; 53 male and 77 female) who were diagnosed with monocular idiopathic macular hole and underwent surgical treatment were enrolled in this study. Among the operated eyes, there was no significant difference in the preoperative axial length or IOP between phakic and pseudophakic eyes. However, patients with phakic eyes were younger than those with pseudophakic eyes (*p* = 0.035). Among the contralateral eyes, the preoperative axial length was significantly shorter in pseudophakic eyes (*p* = 0.019, Table 1).

**Table 1:** Preoperative patient data.

		Total	Phakic	Pseudophakic	*p*-value*
**No.**	MH eye	130	84	46	
	Contralateral eye	130	78	52	
**Mean age (y)**	MH eye	67.43±8.15	66.54±7.70	70.68±9.02	0.035
	Contralateral eye	67.43±8.15	66.68±7.82	69.12±8.70	0.182
**Axial length (mm)**	MH eye	23.23±1.64	23.24±1.66	23.18±1.56	0.695
	Contralateral eye	23.23±3.64	23.26±1.28	23.14±2.46	0.019
**IOP (mmHg)**	MH eye	14.98±2.95	15.09±2.78	14.61±3.54	0.596
	Contralateral eye	14.78±3.15	14.96±2.71	15.05±3.49	0.696

Values are presented as mean ± standard deviation, unless otherwise indicated.

IOP = intraocular pressure, MH = macular hole.

* analyzed using the Mann-Whitney U test for comparison between phakic and pseudophakic eyes.

On postoperative day 2, at least 80% of the vitreous cavity was found to be filled with gas in each patient and the mean IOP was increased compared with the preoperative values in both eyes: from 14.98±2.95 mmHg to 16.82±3.12 mmHg in operated eyes and from 14.78±3.15 mmHg to 16.27±1.87 mmHg in contralateral eyes. However, the difference was significant only in the operated eyes (*p* = 0.039). The IOP increase was significantly greater in patients who received anti-glaucoma eye drops than in those who did not receive an IOP-lowering drug, both in the contralateral (*p* = 0.045) and operated eyes (*p* = 0.031, Table 2).

**Table 2:** Comparison of IOP in both eyes between the untreated group and the IOP control group according to the chronological change after operation.

		Postoperative day 2 (mmHg)	Postoperative day 7 (mmHg)		ΔIOP (mmHg)	ΔIOP (%)	*p*-value*
**Macular hole eye**	Total	16.82±3.12		15.57±6.10			0.341
	Untreated (n = 82)	14.31±2.84		14.46±4.55	0.22±3.24	4.15±2.86	0.452
	Treated (n = 48)	19.04±3.11		18.94±8.62	1.05±4.21	8.43±5.24	0.136
***p*-value**^**†**^		0.031		0.005	0.067	0.124	
**Contralateral eye**	Total	16.27±1.87		14.40±4.14			0.021
	Untreated (n = 82)	14.12±2.85		13.82±3.31	-0.65±2.53	-4.84±2.19	0.084
	Treated (n = 48)	18.05±2.94		16.13±5.72	-0.89±5.05	-5.41±2.81	0.041
***p*-value**^**†**^		0.045		0.033	0.549	0.588	

Values are presented as mean ± standard deviation, unless otherwise indicated.

IOP = intraocular pressure.

* determined by an independent student *t*-test for comparison between postoperative day 2 and postoperative day 7

^†^ determined by paired *t-*test for comparison between treated eyes and untreated eyes based on the postoperative day.

**Fig 1:**
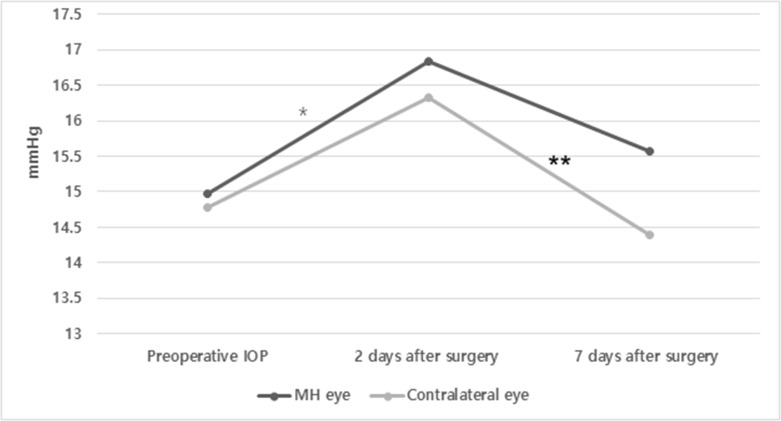
Comparison of the IOP in both eyes according to the chronological change after surgery. * There was a significant difference in the IOP between baseline and 2 days after surgery in operated eyes, paired *t*-test; *p* = 0.039. ** There was a significant difference in the IOP between 2 days and 7 days after surgery with FDP in contralateral eyes, paired *t*-test; *p* = 0.021. The IOP at 7 days after FDP was significantly different between operated and contralateral eyes (15.57±6.10 mmHg in operated eyes and 14.40±4.14 mmHg in contralateral eyes), paired *t*-test; *p* = 0.039. IOP = intraocular pressure; MH = macular hole; FDP = face-down positioning. Data are presented as the mean ± standard deviation.

On postoperative day 7, every patient had sufficient gas volume to cover the macular hole; no one needed additional gas injection; there was a significant decrease in the IOP compared with that on postoperative day 2 in the contralateral eyes (*p* = 0.021, Fig 1). At this time point, the mean IOP was significantly lower in the contralateral than in the operated eyes (*p* = 0.039, Fig 1). In all patients who received anti-glaucoma treatment, the mean IOP decreased in both eyes, but the difference was significant only in the contralateral eyes (*p* = 0.041, Table 2).

Among contralateral eyes, compared with the preoperative values, the mean IOP was increased on postoperative day 7 in eyes with an axial length less than 23.0 mm, but there was no significant difference (*p* = 0.343), whereas in eyes with an axial length of 23.0 mm or more, there was a statistically significant decrease in the IOP after FDP (*p* = 0.042). Furthermore, the mean IOP in contralateral eyes on postoperative day 7 was increased in phakic eyes with an axial length less than 23.0 mm, but without a statistically significant difference (*p* = 0.582, Table 3). However, in phakic eyes with an axial length of 23.0 mm or more, the mean IOP statistically significantly decreased from 14.76±2.53 mmHg to 14.09±3.67 mmHg after FDP (*p* = 0.048). In phakic eyes, the preoperative IOP was not statistically different according to the axial length (*p* = 0.234). However, the postoperative IOP was significantly lower when the axial length was 23.0 mm or longer (*p* = 0.039). The IOP of pseudophakic eyes with an axial length of 23.0 mm or more also decreased postoperatively, but there was no statistical difference (*p* = 0.621). In comparing the IOP in pseudophakic eyes according to the axial length, there was a significantly higher preoperative and postoperative IOP in eyes with an axial length of more than 23.0 mm (*p* = 0.003 and 0.005, respectively).

**Table 3:** Comparison between preoperative and postoperative IOP values at 7 days after maintaining FDP in the contralateral eyes according to the lens status and axial length.

		Preoperative IOP (mmHg)	Postoperative IOP (mmHg)	ΔIOP (mmHg)	*p*-value*
				ΔIOP (%)	
**Total**		14.78±3.15	14.40±4.14	-0.54±3.36	0.253
				-2.53±2.41	
**Axial length**					
** <23 mm (n = 61)**		14.61±3.21	14.69±4.87	-0.20±3.51	0.343
				-0.20±2.44	
** ≥23 mm (n = 69)**		15.33±3.11	14.65±3.69	-0.68±3.18	0.042
				-3.44±2.25	
***p*-value**^**†**^		0.231	0.846	0.576, 0.584	
** Lens status**					
** Phakic (n = 78)**		14.76±2.91	14.48±4.27	-0.50±3.35	0.065
				-2.49±2.49	
** Pseudophakic**		14.85±3.66	14.21±3.87	-0.64±3.42	0.356
**(n = 52)**				-2.61±2.50	
** *p*-value**^**†**^		0.998	0.990	0.735, 0.805	
**Phakic (n = 78)**	<23 mm (n = 36)	15.11±3.28	15.42±5.18	-0.08±3.73	0.582
				1.02±2.56	
	≥23 mm (n = 42)	14.76±2.53	14.09±3.67	-0.67±3.27	0.048
				-3.84±2.21	
** *p*-value**^**†**^		0.234	0.039	0.494, 0.358	
**Pseudophakic (n = 52)**	<23 mm (n = 32)	13.23±2.65	12.70±3.33	-0.54±2.90	0.474
				-3.13±2.31	
	≥23 mm (n = 20)	17.73±4.20	17.00±2.83	-0.73±2.94	0.621
				-1.78±1.68	
** *p*-value**^**†**^		0.003	0.005	0.995, 0.910	

Values are presented as mean ± standard deviation, unless otherwise indicated.

IOP = intraocular pressure, FDP = face down positioning.

* determined by an independent student *t-*test for comparison between preoperative and postoperative IOP.

^†^ determined by a paired *t*-test for comparison based on axial length (23.0 mm) between phakic and pseudophakic eyes.

Among the 82 patients who did not receive anti-glaucoma eye drops, there was no significant difference between the preoperative and postoperative IOP values according to the axial length in both eyes. However, in the contralateral eyes, the IOP of pseudophakic eyes was significantly higher than that of phakic eyes when the axial length was 23.0 mm or more (*p* = 0.003, Table 4).

**Table 4:** Change in the intraocular pressure in both eyes in patients without anti-glaucoma treatment.

		Preoperative IOP (mmHg)	Postoperative day 2	Postoperative day 7	*p*-value*
			IOP (mmHg)	IOP (mmHg)	
**Macular hole eye**		15.01±3.12	16.82±3.12	14.93±4.58	0.425
**AL<23.0mm**	Total	15.14±2.99	15.45±1.14	14.92±4.83	0.534
	Phakic	14.40±3.06	14.62±2.88	13.88±3.07	0.124
	Pseudophakic	13.75±4.27	16.28±5.26	13.00±1.83	0.088
	*p*-value^†^	0.491	0.062	0.537	
**AL≥23mm**	Total	14.86±2.65	14.89±2.41	14.94±4.34	0.514
	Phakic	14.76±2.66	14.21±2.35	14.80±4.48	0.427
	Pseudophakic	15.33±2.80	15.13±1.84	15.67±3.88	0.625
	*p*-value^†^	0.493	0.354	0.756	
**Contralateral eye**		14.63±2.54	16.27±1.87	14.07±3.11	0.124
**AL<23.0mm**	Total	14.38±3.15	14.35±4.23	13.89±3.05	0.262
	Phakic	14.82±3.25	15.13±3.74	14.35±2.86	0.134
	Pseudophakic	13.23±2.65	13.37±3.22	12.69±3.33	0.264
	*p*-value^†^	0.138	0.084	0.141	
**AL≥23mm**	Total	14.87±2.72	15.11±2.14	14.24±3.62	0.221
	Phakic	14.45±2.61	14.87±3.65	13.65±3.63	0.374
	Pseudophakic	16.71±2.27	15.35±4.19	16.86±2.27	0.365
	*p*-value^†^	0.053	0.093	0.003	

Values are presented as mean ± standard deviation, unless otherwise indicated.

IOP = intraocular pressure, AL = axial length.

*Kruskal-Wallis test for comparison of preoperative and 2- and 7-day postoperative intraocular pressure in phakic and pseudophakic eyes

^†^: Wilcoxon sign rank test for comparison between phakic and pseudophakic eyes.

## Discussion

In this study, we investigated the alterations in the IOP not only in the operated, but also in the contralateral eyes over 7 days after surgery followed by FDP, as well as the factors affecting the IOP change in the unoperated contralateral eyes. Maintaining FDP after gas injection can promote retinal adhesion and closure of the macular hole [12]. However, FDP may cause pupillary blockage due to anterior movement of the crystalline lens, which may lead to acute angle-closure glaucoma [13]. Grant et al. [14] reported that an increase in the IOP of 5.2–7.6 mmHg could occur during FDP, while Han et al. [15] reported that the IOP increased to more than 30 mmHg in 35.6% of patients after PPV with intravitreal gas injection and that FDP introduces a possibility of IOP increase. Chen [16] noted that the IOP elevation is transient, with the highest mean values at 2–4 hours postoperatively, and lasts for up to 7 days after surgery. In agreement with these previous findings, in our study, there was an increase in the IOP in both eyes until 2 days after surgery.

In the present study, the IOP increased after surgery in both eyes; however, the degree of elevation was higher in the operated eyes. There was a significant increase in the IOP in operated eyes between baseline and postoperative day 2, which seems to be due to the effect of surgery itself, such as corneal edema or postoperative inflammation. In addition, as noted in previous studies [17–19], the IOP increase in surgically treated eyes could be attributed to the influence of FDP. Furthermore, the IOP significantly decreased between postoperative days 2 and 7 in both eyes, but the degree of descent was higher in the contralateral eyes. From postoperative day 2, anti-glaucoma eye drops were applied to operated eyes when the IOP exceeded 22 mmHg. Previous studies have reported that the beta-blocker used to control IOP elevation in operated eyes may regulate IOP not only in the operated, but also in the contralateral eyes [20, 21]. This is thought to be due to the anti-glaucoma effect of the eye drops, which manifests in both eyes when they are used on either unilateral eye [22].

Further, we found that the IOP was elevated at postoperative day 7 compared with the preoperative values in the operated eyes; however, there was no significant difference. Moreover, there was no IOP increase in the contralateral eyes. In addition, there was a significant difference in the IOP between the two eyes at 7 days after FDP, while there was no difference before surgery. In this study, IOP increase was not observed in the contralateral eyes at postoperative day 7, which differed from the findings of previous studies [23, 24] that reported that IOP changes were most affected by changes in the suprachoroidal pressure induced by changes in body position. However, the changes in the IOP according to the posture were not significantly greater in normal eyes compared with those in eyes with glaucoma [25]. In addition, in normal eyes, it has been reported that the difference in the IOP according to the posture was within the range of 0.3–6.0 mmHg on average [26], but this difference varies from study to study [27]. Therefore, the absence of elevated IOP even after 7 days after FDP in the treatment-naïve contralateral eyes shows that body positioning may not have a profound influence on the IOP in treatment-naïve eyes. However, we measured the IOP after patients remained in the sitting position for 5 minutes and included patients who used anti-glaucoma eye drops from 2 days after surgery, which might have affected the results. Furthermore, the reason why the IOP increased until 2 days after surgery and decreased thereafter was considered to be that FDP was well maintained until the second day after surgery. However, patients may find it difficult to maintain prone position as Ellis and Baines [28] reported that 54% of the patients had difficulty maintaining FDP. In such cases, patients should be positioned close to either lateral decubitus position, as it has been reported in a previous study that the IOP rises less in such position [29].

We further investigated the factors affecting the IOP in the contralateral eyes by examining the difference in the IOP according to the axial length and lens status. In patients with an axial length less than 23.0 mm, which can cause angle-closure glaucoma [30, 31], the IOP was increased after FDP, but without a significant difference. In patients with an axial length of 23.0 mm or more, there was a significant decrease in the IOP 7 days after FDP. The possible reason for this reduction in IOP after FDP in the contralateral eyes with an axial length of 23.0 mm or more could be that the anterior chamber angle was reopened when the body position was changed to measure the IOP, resulting in a relatively increased anterior chamber depth, leading to the IOP being restored to its baseline values. Thus, the reason is thought to be the increase in space caused by the posture change, which may buffer the blood flow alteration. With respect to the lens status, in phakic eyes, there was no statistical difference in the preoperative IOP according to the axial length, but the postoperative IOP was significantly lower in eyes with an axial length of more than 23.0 mm. Likewise, when maintaining FDP, there might be an increase in IOP, even in the contralateral eyes, and the effect seemed to be greater when the axial length was shorter in this study. In addition, the change in the IOP between pre- and post-surgery was significantly greater in phakic contralateral eyes with an axial length longer than 23.0 mm. In this study, there was no significant difference in the IOP between phakic and pseudophakic eyes, both among the operated and the contralateral eyes.

We also found a significant decrease in the IOP after FDP in phakic eyes. The positional IOP change has been reported to be more significant in phakic than in pseudophakic eyes [32]. We observed no significant change according to the posture change in pseudophakic eyes, but there was a significant decrease of IOP in phakic eyes with an axial length of 23.0 mm or more. This might be due to the effect of lens vault on the anterior chamber depth, which causes a possibility for pupillary block, as reported previously [33]. However, the decreased IOP after FDP was thought to be due to the effects of the long axial length, which served as a buffer, and the anti-glaucoma eye drops. Although anti-glaucoma eye drops were applied only to the operated eyes, the IOP in the contralateral eyes was also significantly decreased at postoperative day 7 compared with that at postoperative day 2. This effect was thought to have contributed to lowering the contralateral IOP in cases where the axial length was longer than 23.0 mm, as well as to the absence of IOP elevation despite the FDP. After maintaining FDP, the IOP may be elevated. In the case of a long axial length, where there is a larger range of buffer action, and in phakic eyes within a larger range of IOP change, there was no IOP increase when in a sitting position and with the use of anti-glaucoma eye drops. On the other hand, in case of a short axial length, the FDP may lead to increased IOP.

The limitations in this study include the small number of enrolled patients and the lack of angle evaluation by using anterior segment OCT or gonioscopy. In addition, we could not confirm compliance with FDP, and the follow-up period was short. Further research regarding whether IOP can be used as indirect assessor of compliance to FDP is also warranted.

In conclusion, based on a short-term follow up, FDP after PPV with intravitreal gas tamponade for the treatment of idiopathic macular hole may cause IOP elevation not only in the operated, but also in the contralateral eyes; the IOP increase in contralateral eyes is temporary and less than that in operated eyes and is not maintained until 7 days after surgery; the IOP change seems to be affected by axial length and lens status.
